# Measuring social and emotional learning implementation in a research-practice partnership

**DOI:** 10.3389/fpsyg.2023.1052877

**Published:** 2023-07-26

**Authors:** Nickholas Grant, Joanna L. Meyer, Michael J. Strambler

**Affiliations:** Division of Prevention and Community Research, Yale School of Medicine, New Haven, CT, United States

**Keywords:** implementation, social and emotional learning (SEL), researcher practitioner partnerships, implementation science, measurement

## Abstract

The measurement of social and emotional learning (SEL) implementation is a critical part of enhancing and understanding the effects of SEL programming. Research has shown that high-quality SEL implementation is associated with social, emotional, and academic outcomes. Schools achieve these outcomes in part through organizational practices that emphasize ongoing communication, collaboration, coordination, shared decision making, and strategic planning, processes that are ideally informed by evidence. The application of implementation science to SEL has advanced our understanding of the role of implementation in achieving student outcomes. However, the development of practical approaches for measuring and supporting SEL implementation have lagged behind work on measuring student SEL outcomes. Research-practitioner partnerships (RPP), long-term, mutually-beneficial collaborations geared toward identifying problems of practice and testing solutions for improvement, are a promising means for addressing this important gap. Though implementation science and RPPs have complementary aims, there has been limited attention to the integration of these approaches in the context of SEL programming. The goal of this paper is to offer practical strategies for measuring and using SEL implementation data in schools, using the example of an RPP that used implementation science practices to guide SEL implementation. We give special attention to structures that can support the collection and use of implementation data to improve practice, as well as considerations around developing measures, considering trade-offs of data collection decisions, and conducting data analysis.

## Introduction

When education practitioners implement a social and emotional learning (SEL) approach, they usually are hoping to enhance students’ social and emotional skills. Although there is a great deal of evidence on the impact of SEL programs on a range of student outcomes, SEL practices and the contexts in which they are implemented vary widely (Durlak et al., 2011; Cipriano et al., 2022). Therefore, in most cases, we cannot assume that the effects of a given SEL approach will be the same as the evidence from prior studies. In short, to know whether SEL practices “work” in a specific case, we first need to know about what was implemented, how much of it was provided, and how well it was delivered. However, this essential step in understanding effectiveness if often overlooked and the development of SEL implementation measurement tools has been far outpaced by measures of SEL skills and school climate. As a result, much less is known about questions such as: How much of a program needs to be implemented to see meaningful effects? Which aspects of programs are most associated with effects? Perhaps more importantly, the lack of use of SEL measures in school settings among school staff makes it challenging for schools to monitor the progress of their implementation and to act on ways of improving it. It is this last point on which we place the greatest emphasis in this paper—how SEL implementation measures can be developed and used in efficient ways to support SEL practices.

The slower growth of SEL implementation measures for school use is not for a lack of emphasis from researchers, as it is well-known that the role of implementation is central to understanding program effectiveness. In fact, for decades, there has existed a sub-field of implementation science dedicated to understanding and ensuring strong program and intervention implementation (Bauer and Kirchner, 2020). Implementation science is “the scientific study of methods to promote the systematic uptake of research findings and other evidence-based practices into routine practice, and, hence, to improve the quality and effectiveness of health service” (Eccles and Mittman, 2006). Rather than solely focusing on the impact of an evidence-based intervention on outcomes, implementation science tends to focus on measuring the impact of implementation practices on intervention effectiveness in “real-world” settings (Bauer et al., 2015). These intervention practices evaluated may include program fidelity, quality of delivery, dosage, participant responsiveness or engagement, program differentiation, monitoring of comparison/control conditions, adaptation, and program reach, all of which are important when evaluating the strengths of and barriers to implementation (Durlak and DuPre, 2008).

### Implementation science and social and emotional learning

In the SEL field, researchers have also stressed the importance of implementation when evaluating social and emotional learning (SEL) programs (Meyers et al., 2012; Durlak, 2016; Oberle et al., 2016). Many of these arguments emphasized the importance of studying how implementation strategies are executed to provide information about processes (e.g., school resources and values, decision making processes, team and school staff responsibilities for evaluation, and teachers and staff attitudes) that helped promote implementation success. Further, studies of SEL point to the importance of certain implementation characteristics promoting outcomes in students. For example, a 2011 meta-analysis found four qualities of effective SEL programs: (1) sequenced training approach, (2) active forms of learning, (3) focused and adequate time spent on skill development, and (4) explicit learning goals (Durlak et al., 2011). In general, evaluating these aspects of implementation quality can encompass three forms, including (a) a process evaluation in which there is simply an observation and collection of data related to characteristics of a program either before, during, and/or after it is been implemented; (b) a formative evaluation in which data are collected and shared with the implementation team in order to improve and modify processes of implementation; or (c) a summative evaluation in which data are collected to study the impact of the implementation strategies on program outcomes (e.g., rates or quality improvement of an program; Bauer et al., 2015). In this paper, we primarily focus on ways in which implementation measures can be used in formative ways, but we also address their use in summative evaluation.

At the core of implementation, science is an over-arching goal: to bridge the gap between prevention research and practice by way of developing and evaluating evidence-based interventions and enhancing their use (Chambers, 2012). One framework that illustrates these processes is the Interactive Systems Framework (ISF), which includes three core systems that co-function to improve dissemination and implementation practices: (a) the Prevention Synthesis and Translation System, (b) the Prevention Support System, and (c) the Prevention Delivery System (Wandersman et al., 2008). The Prevention Synthesis and Translation System involves gathering, synthesizing, and translating research literature for practitioner use; the Prevention Support System involves providing innovation-specific support (i.e., intervention related training and providing information and technical assistance with intervention goals) and general support with building the organizational infrastructure and support; and the Prevention Delivery System involves implementing the service activities planned after building capacity. In the example presented in this paper, we discuss what might be considered yet another more overarching framework for supporting implementation—research-practice partnerships (RPPs).

In the context of SEL programs, there are other key implementation-related questions that need to be addressed such as: How much of a program needs to be implemented to see meaningful effects? Which aspects of SEL programs are most associated with effects? Perhaps, more importantly, the lack of practical SEL measures for use in school settings by school staff makes it challenging for schools to monitor the progress of their implementation and to make improvements as needed. It is this last point that we place the greatest emphasis on in this paper—how SEL implementation measures can be developed and used in efficient ways to support SEL practices. We demonstrate these measures using an example of a research-practice partnership (RPP) that used implementation science practices to guide the implementation of SEL in the Bridgeport Public Schools. In the following sections of the paper, we first briefly define and explain the purpose and practices of implementation science. Lastly, we define and outline a framework for RPPs and discuss the implications for implementation science methods within SEL program development.

### Research-practice partnerships

Research-practice partnerships (RPPs) are long-term collaborations between researchers and practitioners that aim to improve education by conducting mutually beneficial research (Coburn et al., 2013; Farrell et al., 2021). RPPs bring together stakeholders from the fields of education research, policy, and practice—fields that are sometimes siloed—to engage the diverse expertise of these stakeholders. RPPs use a variety of strategies to manage the challenges of working in collaboration, including power dynamics that arise from differences in professional backgrounds, individual perspectives, organizational cultures, inter-organizational politics, and much more (Denner et al., 2019; Farrell et al., 2021; Yamashiro et al., 2023). According to a review by Phelps (2019) of 56 studies on challenges in research-practice partnerships in education, building *organizational infrastructure* (e.g., defining roles, decision-making processes, and communication strategies), *shared meaning* (i.e., identifying shared values and understanding of goals), and *trusting relationships* (e.g., favoring equality over hierarchy, respecting the value of diverse contributions) are essential in RPPs.

The guiding principles inherent in the ISF framework align well with the research-practice partnership model. First, ISF posits that research and practice should mutually build upon one another through using scientific literature and evidence-based research methods. Secondly, the ISF invites shared decision-making and collaboration, communication, and strategic planning and coordination among all parties involved in the dissemination and implementation of the intervention (Wandersman et al., 2008; Chambers, 2012). Regarding this latter point, the ISF proposes that multiple parties (i.e., researchers, prevention practitioners, funding agencies, and support agencies) be involved and utilize their scientific knowledge and expertise to (1) understand the capacity required to deliver a specific service and (2) engage in data driven practices to build organizational capacity to promote an intervention’s success (Wandersman et al., 2008; Chambers, 2012).

### RPPs and implementation science in social–emotional programming

The principles of RPPs and implementation science are especially useful within education partnerships that aim to promote SEL competencies among students, such as self-awareness, self-management, social awareness, relationship skills, and decision-making skills that are especially useful for supporting developmental transitions into adulthood (Oberle et al., 2016). Historically, schools have primarily focused on academic outcomes and performance, however, schools have been increasingly integrating SEL programming given its connection with improvements in academic performance, student conduct, school climate, peer relationships, and teacher well-being (Durlak et al., 2011; Oberle et al., 2016; Herrenkohl et al., 2020).

Though impactful, the process of adopting and implementing SEL programming school-wide can be challenging; without buy-in from teachers, school staff, and district leaders, SEL practices and policies will be unsustainable and difficult to implement (Herrenkohl et al., 2020). An RPP can help to address these challenges if it attends to the strategies identified above: building *organizational infrastructure* (e.g., the availability of school resources to assist in coordinating and communicating about SEL), *shared meaning* (i.e., establishing values and goals related to SEL that are shared by stakeholders throughout the research and practice organizations), and *trusting relationships* (i.e., teachers’ perception that SEL programming is in their best interest, district and school leaders’ belief that they will benefit from partnering with researchers, and researchers valuing the expertise of practice-side partners).

Research-practitioner partnership approaches have also been used to enhance the fidelity and sustainability of SEL practices (Ackerman and Skoog-Hoffman, 2020). One notable example of a large scale SEL-related RPP is the Collaborative Districts Initiative of the Collaborative for Academic, Social, and Emotional Learning (CASEL). In 2011, CASEL began partnering with eight large school districts to support and study high-quality SEL implementation. For example, the CASEL-Lowell partnership aimed to understand how to integrate and leverage SEL programming in elementary math classes in order to support teachers (Ackerman and Skoog-Hoffman, 2020). Their collaboration yielded insights indicating that the practices were vital for fostering equitable learning and development for children from diverse backgrounds.

### Purpose of the current paper

The main goal of this paper is to offer practical strategies for measuring and using SEL implementation data in schools that draw upon practices drawn from implementation science and the RPPs. Throughout the paper, we highlight examples from an RPP focused on the implementation of a social and emotional learning initiative in an urban district. After providing context about the partners and the SEL initiative, we discuss ways in which researchers and practitioners can work together to develop implementation measures and structures that facilitate the sustainable collection and utilization of data. We also discuss methodological trade-offs concerning data privacy and data linking important considerations when analyzing collected data and reporting findings.

## The Bridgeport public schools SEL initiative

We begin the remainder of this paper by describing the Bridgeport Public Schools (BPS) SEL Initiative, which grew out of the Yale-BPS SEL Partnership, a research-practice partnership that began in 2013 as a collaboration between BPS, The Consultation Center at Yale School of Medicine (YSM), and Yale Center for Emotional Intelligence. This partnership aimed to build the social and emotional skills of BPS administrators, teachers, staff, students, and their families. At the start of the Yale-BPS SEL Partnership in the 2013–14 school year, the school district was serving 19,231 students enrolled in grades PK-12 at 28 elementary/middle schools, seven high schools, one early childhood center, and two alternative schools. Approximately 49% of students identified as Hispanic or Latino of any race, 37% of students identified as Black or African American (and not Hispanic or Latino), and 100% of students qualified for free or reduced-price lunch [Connecticut State Department of Education (CSDE), 2023]. In 2014, Bridgeport was the Connecticut’s most populous city with about 148,000 residents; it had an estimated median household income of approximately $43,000 for 2012–2016 (compared to approximately $72,000 for the state), making it one of the poorest cities in the state (Connecticut Data Collaborative, 2023). In addition, the school district faced challenges related to discipline concerns and low academic performance. In 2013–2014, the BPS rates of chronic absenteeism and suspensions were more than double the statewide rates [Connecticut State Department of Education (CSDE), 2023].

The Yale-BPS SEL partnership included a collaboration with a diversity of stakeholders/practitioners with expertise in school policy and practice. For instance, members of the university team worked directly with representatives from the district leadership team (e.g., superintendents, assistant superintendents, and SEL coordinators) and school leaders (principals and assistant principals). As the practice partner, the district led SEL decision-making and managed the implementation of SEL programming. As the research partner, The Consultation Center supported SEL implementation and continuous improvement through data collection, analysis, and reporting, serving as a formative and summative evaluation partner over the first 5 years of the partnership.

The Yale-BPS SEL Partnership began in the summer of 2013 with a 5-year grant from the Tauck Family Foundation awarded to The Consultation Center. The funding was initially intended to support SEL implementation in one school with the intention of gradually scaling up the work to include 3–4 schools. However, when the superintendent left his role mid-way through the 2013–14 school year, the new superintendent was so enthusiastic about the work that she charged the partnership to expand the work districtwide; additional funding was sought and acquired to do so. The overall goal of the partnership was to: (a) promote learning, healthy interpersonal relationships, and sound decision-making; (b) foster safe, supportive, and respectful classrooms and schools; (c) utilize measures relevant to these goals that can be used to measure progress, gage impact and guide improvements; and (d) create a model for school improvement that actively engages all stakeholders (Strambler and Meyer, 2018). Table 1 describes the focus of the 5-year Yale-BPS SEL Partnership; related materials are available on Open Science Framework at https://osf.io/nwzrs/. In 2018, The Consultation Center transitioned to an as-needed consultative role and the Bridgeport Child Advocacy Coalition (BCAC) at RYASAP became the partnership’s co-leader alongside the district. As of the last quarter of 2022, the SEL initiative is still in place and has persisted across four superintendent transitions.

**Table 1:** Timeline for the Yale-BPS SEL Partnership.

School year	Focus
2013–2014	Strategic planning, capacity building, and RULER pilot
2014–2015	Leadership development and capacity building at district and school level
2015–2016	Integration of SEL at all schools
2016–2017	Continued SEL implementation and evaluation
2017–2018	Sustainability of SEL implementation

A description of each year’s activities is available on Open Science Framework at https://osf.io/kdh5t.

### RULER

The BPS SEL initiative began with the introduction of RULER, which was developed at the Yale Center for Emotional Intelligence (Brackett et al., 2019). Unlike other SEL frameworks that focus on various inter-and intra-personal competencies (e.g., CASEL; see Blyth et al., 2018), RULER is an evidence-based approach to social and emotional learning designed to enhance emotional intelligence in educators and students. RULER stands for the five key emotion intelligence skills, this approach intends to promote: recognizing, understanding, labeling, expressing, and regulating emotions. The RULER approach relies on first teaching educators (principals, teachers, and school staff) to appreciate the significance of their own and their students’ emotions. The RULER approach asks educators to value the skills of recognizing, understanding, and managing emotions; to learn and model these skills; and to support, teach, and encourage students to develop these skills. Instead of being taught as a separate lesson or set of activities, RULER is designed to be integrated into the everyday routine of teaching and learning, by infusing it into classroom practices and the curriculum. For example, the RULER *feeling words* approach lays out a process for building students’ emotion vocabulary that can be applied to fiction or non-fiction texts in the curriculum. As described by Brackett et al. (2019), RULER also provides four *anchor tools* that can be used across the day and the school year to support the development of social–emotional skills: the *Classroom Charter*, *Mood Meter*, *Meta-Moment*, and *Blueprint*. For example, the RULER *Mood Meter* is a tool that teachers and students can use together or independently to develop awareness of their emotions and how to shift among emotions to enhance learning.

Yale Center for Emotional Intelligence typically uses a train-the-trainer model, in which a school or district identifies a small group of school or district personnel (known as a RULER Implementation Team, RIT) to attend RULER trainings conducted by YCEI. A school RIT typically includes at least three people: a school leader (principal, assistant principal, dean of students, etc.), a school counselor or social worker, and at least one teacher. When they return to their school, the members of an RIT are expected lead professional development for their colleagues and support RULER implementation. RITs are encouraged to first implement RULER among their faculty before classroom implementation begins. In Bridgeport, the districtwide SEL initiative began with a readiness/leadership development year, when all district and school leaders participated in a series of workshops, meetings, and individual coaching focused on the development of emotional-intelligence leadership mindsets and skills *before* school teams began RULER training. The BPS SEL initiative also had the benefit of a full-time SEL coordinator, an experienced educator with RULER training, who provided focused support to school teams starting when the teams began RULER training.

In practice, schools vary in their readiness to implement RULER, which may relate to school administrators’ willingness or ability to dedicate professional development time to RULER, the preparedness of RIT members to train their colleagues, or teacher buy-in. Some schools launch RULER quickly and with fidelity, while other schools are slower to introduce the approach to their teachers and ultimately, their students. As noted above, the implementation quality for any intervention is likely to influence the intervention’s effects. The central goal of the Yale-BPS SEL Partnership was to monitor SEL implementation across schools to identify areas of strength and areas of need, so that resources and support could be allocated appropriately.

## Developing implementation measures

When we set out to develop implementation measures for the partnership, a high priority was placed on measures that were useful, practical, low-burden, and inexpensive. Accomplishing this meant giving special attention to balancing feasibility and rigor. The first step in the process involved holding discussions with district leaders about what data were meaningful to them. To maximize the value of data collection, we discussed which data would not only be valuable for assessing the implementation progress, quality, and signals of impact but could also be useful for other related initiatives in the district. For example, we worked with the district to develop a school climate survey that could inform the districts’ Safe Schools Healthy Students project as well as the SEL initiative. In these discussions, it was crucial to define how the data would be collected, who would use it, for what purposes it would and would not be used, and how interim results would be disseminated. This issue is crucial because data that is collected without being used in meaningful ways by the district is a burden without adequate benefit to the district. Ideally, any SEL-related data collected would be useful at multiple levels—by district leaders, school leaders, school SEL teams, and potentially, by teachers, as each group plays an important role in improving implementation. District leaders can identify resources and supports that schools need for high-quality SEL implementation. School leaders must provide the supports and vision to implement SEL practices. School SEL teams can serve as resources for one another, especially those that have consistently strong implementation. And teachers are essential as the main implementers of SEL practices in the classroom.

**Figure 1:**
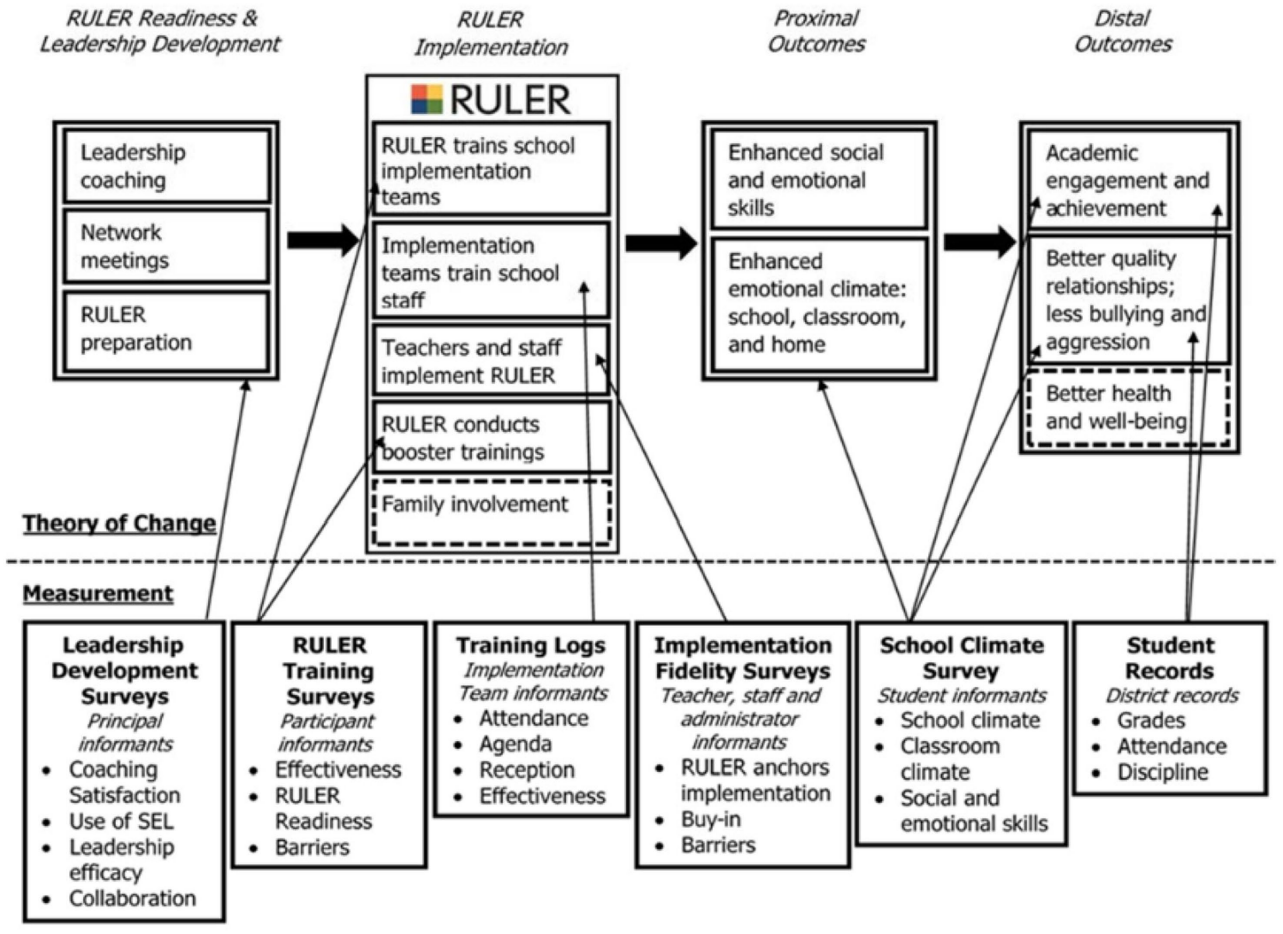
Bridgeport SEL initiative theory of change mapped to measurement tools.

In terms of developing practical measures, one important consideration is the type and nature of the measures used. This is essential because, depending on the type of measure, data collection can be very time-consuming. Because observational measures require a great deal of time and resources to use, SEL implementation data is often collected through teacher self-report measures that ask teachers about the SEL practices that they are engaged in. In selecting these measures, it is important to consider the measures’ sensitivity to change. That is, the ability of the measure to pick up on change of what it is capturing over the period of time that the measure is being used. The same measure that is intended to capture change over a 6-month period may not be sensitive enough to pick up on change over a 2-month period. Thus, it is valuable to use theory to develop or select items that have a chance of changing over the time being assessed. While some established measures provide psychometric information regarding sensitivity, typically this needs to be assessed following the collection of the data by inspecting change scores. If there is very little change, there could be two possibilities—that the measure genuinely did not change or that the measure was not sensitive enough to pick up on the change. If data are collected over multiple time points it provides various time points to examine such change. We used a variety of implementation measures and other measures over the course of the Yale-BPS SEL Partnership to balance rigor, feasibility, and sensitivity to change, and to account for potential bias. Figure 1 depicts the initiative’s theory of change and the measures used at each stage (Strambler and Meyer, 2018). As shown in Figure 1, the partnership developed two types of SEL implementation measures: SEL implementation logs and SEL implementation surveys, which are discussed below, along with other SEL implementation measurement approaches that we considered but did not use. Note that this paper does not discuss the leadership development surveys and RULER training surveys that were used to assess the readiness and training phases, nor does it describe the school climate and SEL student survey and administrative data that we used to assess student outcomes.

### SEL implementation logs

The logs were designed to track SEL-related trainings and activities at the school level in the first 2 years after RULER training. During the final day of RULER training, we met with the school teams to explain the purpose of the SEL implementation log and asked each school’s SEL team to identify a contact person who would be responsible for completing and submitting the school’s SEL log each month. The researchers worked with the SEL coordinators to design this measure to be brief and easy to complete, with the final version of the measure consisting of only six items, two of which were optional open-ended questions. The first item asked the team to estimate how much whole-staff meeting or professional development time the school had spent on each of six RULER topics over the past month, with responses ranging from none to over 60 min. The second question asked the SEL team to rate the knowledge of their school staff regarding the same six RULER topics, with response categories of *Beginning, Progressing*, and *Advanced*. The third and fourth items asked the SEL team to describe any RULER events held for students and parents, respectively, during the past month. The last two questions asked the SEL team to share any comments about how implementation was progressing and what additional supports they needed to support implementation. A copy of the 2016–2017 SEL log is available on Open Science Framework at https://osf.io/h86am. The SEL log was mailed to school contact people monthly as an online survey on the Qualtrics platform, with additional reminders sent to those schools who did not complete the survey on time. At the end of the month, we compiled the results and shared them with the SEL coordinators, so that these district leaders could follow up with individual schools, as needed.

### SEL implementation surveys

As described by Yeager et al. (2013), we needed to manage the level of burden on school personnel while collecting detailed data about on-the-ground implementation that could inform improvement. Classroom observations were impractical given the scale of the district-wide initiative and funding constraints. Instead, we took a more practical approach to measurement that focused on implementation specific related questions that would have more direct implications for service improvement strategies (Yeager et al., 2013); we chose to conduct periodic surveys of school leaders (i.e., principals and assistant principals), teachers, and other district personnel. In close consultation with the RULER developers, SEL coordinators, and other district personnel, we created surveys that asked educators about their perceptions of SEL implementation in their school and district. While developing the survey, we met with our partners several times to discuss potential survey items, in order to ensure we were prioritizing domains of interest, asking questions clearly and efficiently, and keeping the survey to a manageable length. Ultimately, we asked about *quality of implementation, support for program implementation, teacher attitudes, perceived barriers to implementation, principal factors, and professional experience*. Different respondent groups saw different sets of questions, as documented in Table 2. We continued to meet with our partners before and after every survey administration to discuss survey data and potential revisions to survey items. Although we generally sought to keep items consistent to allow for comparisons over time, our partners’ input led to some revisions to improve clarity and address evolving priorities. The 2015–16 through 2017–18 versions of the survey are available at https://osf.io/n8dfy/.

**Table 2:** SEL implementation survey domains by respondent.

Measure	Respondent		
	Teachers	Other staff	Principals/APs
**Quality of program implementation**			
Anchors knowledge	X	X	X
Introduction to anchors	X		
Use of anchors	X	X	X
Integration of anchors			X
Fidelity	X		
Perceived self-efficacy	X	X	X
**Support for program**			
Principal support	X	X	
Internal support	X	X	
External support	X	X	X
**Program receptivity**			
Goodness-of-fit	X	X	X
Participant engagement	X	X	X
Perceived program effectiveness	X	X	X
**Perceived barriers to implementation**			
Barriers			X
**Experience**			
Professional experience	X	X	X

As with the logs, a key decision was how often to administer the implementation surveys to staff. We consulted with our district partners about how many times per year would be feasible while taking into consideration the time staff had available to complete the survey and the other surveys that they were expected to complete a greater opportunity to observe change over time. It is important note that district buy-in was essential to meaningful data collection. Although the district emailed the survey link to all teachers and staff members, response rates were relatively low for the initial survey, and the superintendent and SEL coordinators expressed concern that the data may not be sufficiently representative of the experiences of district personnel as a whole. The superintendent and our team were also concerned that we would not see signals of change in SEL implementation if we only surveyed teachers and staff twice in the fall and spring. We collectively agreed to add a winter survey administration the following year, and we collaborated with our partners to increase response rates in subsequent surveys. For example, the superintendent pointed out that all schools held a monthly staff meeting on the first Wednesday of the month. She asked us to schedule each future survey to launch the day before a monthly staff meeting, and she directed principals to allocate time during those meetings for teachers and staff members to complete the survey. In addition, we agreed that during each survey administration, the research team should share weekly reports showing response rates by school so that the district could follow up with the principals of schools with low response rates to ask them to re-send the survey link to their teachers and staff. These collaborative efforts increased response rates dramatically and increased the confidence of district and school leaders in the value of the data.

### Other potential data collection

While we did not have the capacity to support studying how data were used by the teachers and administrators, this can be an important process for understanding the effectiveness of the data use process. For instance, one option researchers could consider are mixed methods in which the quantitative surveys described herein are paired with qualitative interviews that focuses on how practitioners interpreted and used the data to inform decision-making. Utilizing mixed methods has multiple benefits; combining elements of qualitative and quantitative research methods and analyses allows researchers to clarify and/or develop their research approach to converge, corroborate, expand, or elaborate on research findings (Schoonenboom and Johnson, 2017). Though survey data have the potential to produce evidence that is generalizable to a larger population, the structured format limits the ability to document individuals’ subjective experiences, especially when such experiences do not fit well within constructs assessed by surveys. Interviews and focus groups, however, are very useful when the objective is to understand how individuals construct meaning of what is relevant and salient to them, and descriptive details about context-specific actions within settings (Nowell et al., 2017). Conducting follow-up interviews with teachers, administrators, and district leaders could shed light on how they personally experience the implementation strategies applied. Specifically, qualitative interviews could (a) help discern possible strengths and areas in need of change within implementation strategies, (b) provide multiple perspectives across the leadership hierarchy, which can identify areas of miscommunication and converging and diverging opinions about actions taken; and (c) create discussion of improvement recommendations that are grounded in the practitioners’ experiences.

## Teacher privacy and linking of implementation data

One necessary decision to make when collecting implementation data from school staff is whether to collect the data in a confidential, but identifiable way, or anonymously. From a research perspective, it is advantageous to collect the data in an identifiable way since it allows for individual teachers’ implementation practices to be examined over time; it also allows for teacher-reported implementation data to be linked to student outcomes (assuming these data are accessible). However, educators’ concerns about privacy need to be taken seriously in school-based research, to ensure that educators feel comfortable sharing their perspectives. It is not uncommon for teachers to feel uncomfortable with providing identifiable implementation due to concerns about it being used in an evaluative way rather than a supportive one. Even if the data collector were to use methods to ensure confidentiality, teachers may be understandably skeptical about whether their privacy is protected adequately. Therefore, one is often faced with a tradeoff. If data are collected anonymously, it protects privacy but limits the ability to link implementation data to students’ outcomes. Yet, if data are collected in an identifiable manner, it allows for linking and other data analytic options, but runs the risk of losing the trust of the school staff and potentially biasing educators’ responses toward reporting in ways that they view as more favorable. Especially in a partnership context, if one suspects that a substantial portion of the teaching body is concerned about privacy, the most prudent choice is to collect data anonymously given that trust among partners is essential for all aspects of the work. For the reasons noted above, for the Yale-BPSSEL Partnership, we opted to collect survey data anonymously, where the only identifying characteristics were the teachers’ school and role. While this prevented us from linking teachers’ responses over time and from linking teachers to students to analyze implementation data at the classroom level, we were able to create school-level implementation measures and to link them to student outcome data.

## Summarizing and analyzing implementation data

Once SEL implementation data are collected, there are two broad ways in which the data can be summarized to use for formative purposes—descriptively and statistically. Descriptive summaries (for example, frequency tables), visualizations (for example, frequency plots), or combining items into composites using mean or sum scores, are especially useful for using data continuous improvement purposes. These data can also be organized thematically in ways that are most meaningful to staff members. For example, a self-report measure might involve a collection of items organized around the components of an SEL program. In such cases, decisions might be made about reporting single items under category headings or creating mean and/or sum scores of the items of such items. In general, it has been our experience that when using Likert-type continuous items, means are more interpretable than sum scores.

In the case of the Yale-BPS SEL Partnership, the way we presented data to our partners depended on the audience. As noted above, we provided the SEL coordinators with a tabulation of implementation log responses at the end of the month. The district was not interested in a summary or descriptive statistics for the SEL logs, because the SEL coordinators were using each school’s response to guide their interactions with that school. The monthly report gave the SEL coordinators a snapshot of each school’s progress that the SEL coordinators could use to start conversations and provide tailored supports. For example, if a school’s SEL team reported that they had hosted their second all-staff RULER training, the SEL coordinators could ask about how it went. If a school’s SEL team reported that they had not done anything in the past month, the SEL coordinators could inquire about barriers and offer their support.

For the SEL implementation surveys, which received responses from hundreds of educators, it was essential to summarize and visualize overall responses descriptively and also to share each school’s results with its leaders. For this reason, we communicated results from each survey to our partners in four formats. First, we generated a district-level summary report, which showed frequencies and means for key survey items, to share with our district administrator partners. Second, we generated a school-level report for each school, which included frequency tables and plots for each item, to share with the principal and assistant principal at each school. Third, we generated a district-level detailed report, which showed frequencies and means for key survey items broken out by school and with comparisons over time, to share with our district administrator partners. Finally, we presented survey results to all district and school leaders as part of one of the districts regularly scheduled meetings for administrators. At these meetings, we focused on a small number of key items and discussed how responses were changing over time. For example, we reported the percentage of teachers who said they had used a specific SEL practice with their students in the past week. We typically provided the district-level summary report within 10–14 days of the survey administration, so that the SEL coordinators, the superintendent, and her leadership team could see an overview of the data when it was still quite recent. Although it took more time to produce the detailed reports, we made sure to share them with district and school leaders within 1 month while the results were still relevant. We were usually invited to present at the first administrator meeting after the survey.

Although the Yale-BPS SEL Partnership did not have a process for systematically tracking how district or school leaders received and used data from the SEL survey, we believe it useful to provide some anecdotal evidence about how we built trust and buy-in around the collection and use of data. Our first presentation to the BPS administrative council was during the leadership development year. Our first goal was to explain The Consultation Center’s role within the districtwide SEL initiative that the superintendent had launched the preceding summer. Our second goal was to explain what data would be collected and why. In this initial presentation, we explained that in close collaboration with district leaders, we would use data to know whether we were achieving our goals, to improve professional development programming provided by Yale Center for Emotional Intelligence, and to facilitate evidence-based decision-making. We made it clear that we were not evaluating school leaders or teachers, and we emphasized how we would protect the confidentiality of survey respondents throughout the project. These themes remained central when we presented to the administrative counsel two more times that year and in subsequent years, as well as remaining central in our meetings with the SEL coordinators and superintendent. Over time, we observed greater interest and engagement among school leaders during our presentations and more instances where they approached us with questions in person or via email. We took these interactions as signs of greater trust although we do not have data to this effect.

Meeting with these partners over time also allowed us to build interest in the data we were sharing, especially when we were able to build curiosity. Initially, the SEL coordinators valued qualitative data from the SEL logs more than quantitative data from the SEL survey. We suspected that part of the problem was that while it was challenging to consider each survey item separately, the SEL coordinators found it challenging to interpret or use the reported scale scores. We also suspected that while it was overwhelming for them to review 30 school-level reports, the SEL coordinators were interested in school-level results. To address these perceived concerns and to promote their interest and investment in the data, we designed an experience to help the coordinators interpret and connect with the data. Specifically, we brought three simple bar charts to a meeting with the SEL coordinators, each of which showed the median value by school for one of three items in the “teacher self-efficacy” scale, but the schools were not labeled by name. This approach piqued SEL coordinators’ curiosity as they began looking for patterns to try to guess which school had produced which values. This practice of observing patterns with real data provided a basis for us to discuss the basis for applying these skills more broadly to the full reports we provided. The meeting is memorable because it marked a shift in the SEL coordinators’ investment in the SEL survey as a source of meaningful data. We also aimed to build curiosity in school leaders about their schools’ SEL survey data by presenting district-level results to them at a meeting before they received their school-level reports. We found that sharing the district-level results at these meetings got school leaders excited to receive their individual reports and to see how their school-level data would compare to the district as a whole.

While descriptive summaries can be useful for research purposes, sometimes summarizing the data requires using more sophisticated techniques. For example, when the data are intended to be used for predicting student outcomes from implementation measures. Reporting such findings can be challenging when sharing them with practitioners who usually do not have the research background to interpret technical statistical findings. In such cases like ours, it is necessary to translate findings in a way that is interpretable to practitioners. When conducting analyses focused predicting student outcomes from implementation, we used multilevel confirmatory factor analysis to create school-level measures of implementation, and then used multilevel modeling to examine the relationships between these measures and outcomes. It would have been inappropriate to report such findings as one would for a scientific journal. Instead, in a brief report, we described the goal of these statistical techniques in lay terms and summarized results visually. As shown in the example in Table 3, we use symbols to indicate whether effects were effects were present (positive or negative) or absent (blank) and color-coded these findings to indicate whether they were in the expected direction (green if yes, yellow if in the opposite of the predicted effect). Researchers can increase the level of detail in these types of depictions, such as including regression coefficients and other relevant statistics, depending on the background of the audience to which they are presenting.

**Table 3:** Practitioner-oriented reporting of the statistical association between SEL implementation (as reported by teachers) and school climate outcomes (as reported by students).

	Student-teacher trust		Rules and norms		Emotional climate		Peer support	
*Grade level*	3–5	6–8	3–5	6–8	3–5	6–8	3–5	6–8
Fidelity of Implementation		+		+	+		+	
Support for Implementation					−			
Perceived Effectiveness							−	
*n*	1,941	2,081	1,946	2,085	1,938	2,067	1,941	2,080

Note: Plus signs indicate positive correlations, and minus signs indicate negative correlations. Green shading indicate that correlations are in the expected/favorable direction, whereas yellow boxes indicate counterintuitive/unfavorable effects.

## Approaches for building sustainable implementation data use

Anytime a partnership is established between researchers and practitioner careful attention needs to paid to sustaining the practices implemented. However, a sustainability practice that is often underappreciated is considering the sustainability of data use. Also. while it is common for sustainability to become the focal point toward the end of implementation-supporting resources such as grant funding coming to an end, we argue for the importance of building sustainable data practices from the start. We have found that regular and meaningful opportunities to promote the engagement of partners with data can go a long way for deepening the roots of the partnership and increasing the chances for sustainability—from the development of measures to the collection of data to exploring what the data are saying. It is often the case that education practitioners find the collection and use of data as detached from the “real work” of teaching students. This is in part because researchers are not commonly trained on the nuances of managing the factors associated with building enduring data procedures and practices that work for *practitioners* as opposed to other researchers. In the dissemination space, there are four stakeholders that are important to consider engaging around data: (1) key district leaders; (2) school leaders and their SEL implementation teams; (3) teachers and other school staff; and (4) community partners. Engaging such a broad “web” of stakeholders is especially valuable in urban settings where there is a higher rate of leadership transition at the school and district levels. Sharing valuable information about the progress of SEL implementation with various stakeholders can help keep the partnership engaged and motivated to continue their mission, even in the midst of top-level leadership changes such superintendents.

In Yale BPS SEL Partnership, there were four partnership structures that were developed and utilized to support the dissemination of implementation data. One structure was the establishment of SEL teams at each school as noted above. As depicted in Figure 1, the school-based SEL teams were established prior to SEL implementation during a “RULER-readiness” phase of the initiative’s rollout. These teams, consisting of 4–5 school staffs supported RULER trainings and the monitoring of SEL implementation at the school level. The second structure was an SEL task force, which consisted of district-level members and representatives from community-based organizations and universities. The SEL task force met quarterly to provide updates about the initiative’s progress and opportunities for input from members. The Task Force served as a valuable venue for the evaluation team to provide status reports about SEL implementation across the district and to receive input about improving implementation.

**Figure 2:**
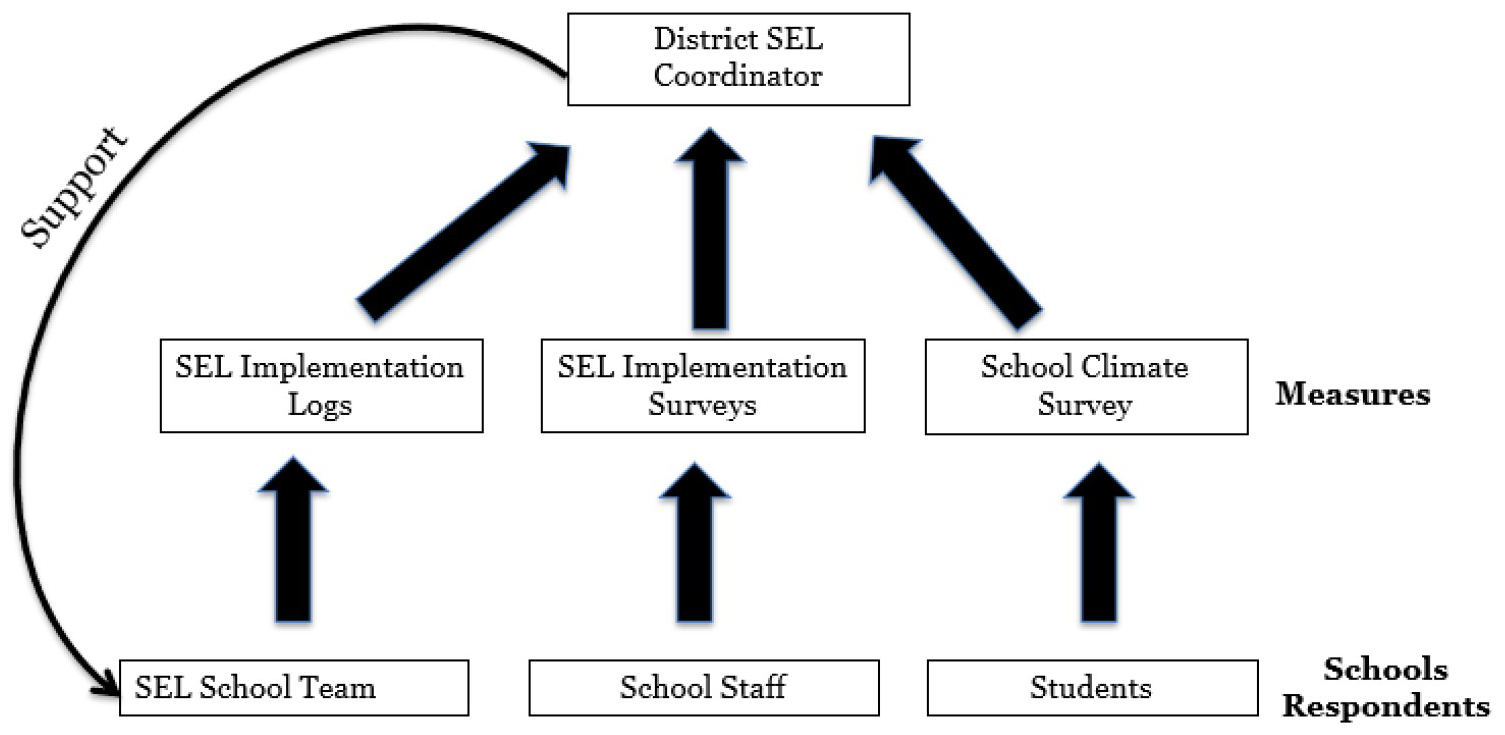
Illustration of data flow and data use for school supports.

The third structure was the establishment of monthly meetings between the university partners and the district partners, specifically the researchers, the RULER developers, the SEL coordinators, and the superintendent. These meetings provided a crucial opportunity for communication and strategic planning and demonstrated the district leadership’s deep commitment to the partnership. The fourth structure for supporting sustainable implementation and data practices was establishing a SEL coordinator position at the district level—a person who is responsible for overseeing and supporting SEL implementation. This role greatly facilitated the use of implementation data at both the district and school levels. The coordinator proved to be instrumental in using the implementation data in ways that were palpably useful. In Figure 2, we depict the flow of data to the coordinator and how the coordinator used it. As shown, the evaluation team would compile implementation data from the SEL team logs and educator surveys in addition to student outcome data and share it with the SEL coordinator, who in turn would use the data to identify where implementation was going well and where it needed more improvement. These data would be used to inform her regular visits to the schools focused on supporting and strengthening implementation.

## Conclusion

Whenever one is interested in studying the effects of SEL, it is also important to consider coupling outcome measures with measures of SEL implementation. Doing so allows one to move beyond understanding whether SEL programming works to understanding why and how it works. Although the development of measures of SEL implementation is lagging measures of SEL outcomes, the field is rapidly growing in this area with implementation being afforded a greater deal of attention. In this paper, we focus on strategies for advancing SEL implementation. First, developing useful measures of SEL implementation that are feasible to use and capture meaningful indicators, provides valuable information to district and school leaders about the progress of SEL implementation. This information is especially helpful for understanding where implementation progress is being made, where more supports are required, and how to make use of effective implementation happening in schools to support the less effective ones. As we discuss in this paper, to make these measures as useful as possible, researchers should be in regular consultation with district and school leaders during the development/selection of measures and the methods for administering them. To ensure that the measures are aligned with the theory of the program that is being implemented, it is also essential that one consult the program’s theory of change, and/or the program developers if possible. When SEL practices are “home grown” by districts or schools, the developers should create a theory of change or logic model that articulates a clear process about the key elements of the practices and how they are anticipated to effect outcomes. In short, the aim of these practice recommendations is to make measures that are useful, practical, and reflective of the theory and mechanisms expected to change outcomes.

We also emphasize the importance of building and maintaining relationships with practitioners in the development and administration of implementation measures. While this is important to do in any context, an especially effective way of doing this is through research-practice partnerships (RPPs) that create opportunities for researchers and practitioners to have ongoing collaborative interactions with each other that are mutually beneficial. In such partnerships, practitioners benefit by expanding their capacity to conduct implementation evaluation and research—a capacity that is often very limited in schools and districts. Practitioners also have opportunities to make valuable contributions to the work such that it reflects what measures that are important to them. For researchers, RPPs can help make the research they care about more relevant and applicable in the “real world.” RPPs can also help advance scientific knowledge by improving our understanding of the nuances of setting features that act to enhance and hinder high quality implementation. Finally, by advancing our knowledge of implementation and connecting them to outcomes, we can improve our understanding of the “active” ingredients most important to impacting outcomes.

## Author contributions

NG contributed to conceptualization of the manuscript, writing of the manuscript, and reviewing. JM contributed to conceptualization of the research and manuscript, carrying out the described research, writing of the manuscript, and reviewing. MS contributed to conceptualization of the research and manuscript, carrying out the research, writing of the manuscript, and acquiring funding. All authors contributed to the article and approved the submitted version.

## Funding

The Tauck Family Foundation supported this research.

## Conflict of interest

The authors declare that the research was conducted in the absence of any commercial or financial relationships that could be construed as a potential conflict of interest.

## Publisher’s note

All claims expressed in this article are solely those of the authors and do not necessarily represent those of their affiliated organizations, or those of the publisher, the editors and the reviewers. Any product that may be evaluated in this article, or claim that may be made by its manufacturer, is not guaranteed or endorsed by the publisher.
